# Development and web-based implementation of a machine learning model for predicting one-year adverse outcomes in acute ischaemic stroke

**DOI:** 10.3389/fmed.2026.1956859

**Published:** 2026-09-18

**Authors:** ChunYu Meng, FeiFei Zuo, Shuai Liu, YuLiang Ji, XingYu Zhu

**Affiliations:** 1 Department of Rehabilitation Medicine, Xuzhou First People's Hospital, Xuzhou, Jiangsu, China; 2 Department of Cardiovascular Medicine, Shuyang Hospital of Traditional Chinese Medicine, Shuyang, Jiangsu, China

**Keywords:** acute ischaemic stroke, machine learning, prognostic prediction, risk stratification model, SHAP interpretability analysis, web-based assessment tool

## Abstract

**Background:**

This study aimed to develop an interpretable and clinically applicable machine learning prediction model to assess the risk of adverse outcomes within one year following acute ischaemic stroke, and to establish a corresponding web-based risk assessment tool.

**Methods:**

A retrospective cohort study included 585 patients with acute ischaemic stroke, randomly allocated into training (70%) and validation (30%) datasets. Recursive feature elimination (RFE) was employed to identify pivotal predictors. Ten machine learning algorithms were constructed and evaluated using area under the receiver operating characteristic curve (AUC), calibration plots, and decision curve analysis (DCA). Model interpretability was enhanced through SHapley Additive exPlanations (SHAP) analysis.

**Results:**

The random forest algorithm demonstrated superior discriminative performance, achieving a validation AUC of 0.870 (95% CI 0.816–0.923). DCA confirmed optimal clinical net benefit with this model. SHAP analysis identified age, D-dimer, and systemic immune-inflammation index as principal determinants of adverse prognosis. A web-based risk calculator was subsequently deployed (https://zxy08887.shinyapps.io/1YPP-AIS-Model/).

**Conclusion:**

This study established a high-performance, interpretable machine learning model capable of accurately stratifying one-year prognostic risk in patients with acute ischaemic stroke. The web-based tool provides a proof-of-concept prototype for individualised risk assessment. However, strict external validation in independent cohorts is required before it can be considered ready for clinical deployment.

## Introduction

As a leading cause of disability and cognitive impairment worldwide, ischaemic stroke accounts for approximately 5.2% of global mortality (1), imposing substantial burdens on healthcare systems and society (2). Consequently, reducing stroke incidence, mortality, and disability has emerged as a pivotal objective in contemporary medicine. Despite notable therapeutic advances, stroke remains the third leading cause of death globally, surpassed only by cardiovascular disease and malignancy (3). Development of accessible and robust prognostic risk stratification tools for early identification of high-risk patients is therefore of paramount clinical importance.

Current prognostic approaches predominantly rely on risk scoring systems and biomarker-based models (4, 5). However, these conventional methods exhibit inherent limitations, notably variable predictive accuracy across diverse populations. Such inconsistency largely stems from reliance on restricted clinical characteristics and demographic variables, inadequately capturing individual patient heterogeneity. Although machine learning (ML) methodologies offer promising opportunities to enhance predictive performance, their clinical implementation encounters substantial obstacles (6). High-performance ML algorithms, particularly deep learning and ensemble methods, often function as “black boxes” owing to their intricate decision-making architectures, precluding intuitive interpretation (7). This opacity impedes clinicians’ and patients’ comprehension of predictive outputs, thereby limiting confidence in and adoption of model-guided clinical decision-making. Within medical practice, model interpretability frequently supersedes predictive accuracy in importance, as clinicians and patients require transparent insight into the rationale and processes underlying predictions.

This study incorporates SHapley Additive exPlanations (SHAP), a sophisticated model interpretation framework, to address the interpretability deficiencies inherent in traditional ML approaches. SHAP, grounded in cooperative game theory, quantifies each feature's marginal contribution across all possible feature coalitions, thereby elucidating individual feature impacts on predictions. This methodology provides multilevel explanatory capacity spanning global to patient-specific interpretations (8, 9), furnishing researchers with powerful tools to comprehend model decision mechanisms whilst identifying pivotal predictive features. Through SHAP, we can analyse and validate ML model outputs at both global and local scales, substantially enhancing transparency in the decision-making process. Furthermore, such interpretability techniques promise to strengthen clinician trust and facilitate clinical adoption, establishing a robust foundation for widespread implementation across diverse clinical contexts.

This investigation systematically applies multiple ML methodologies to evaluate their efficacy in predicting long-term outcomes following ischaemic stroke, employing SHAP and complementary interpretability analyses to elucidate decision mechanisms and key determinants. By integrating conventional clinical parameters with multidimensional prognostic factors, we aimed to enhance both predictive accuracy and generalisability. Subsequently, a practical individualised prognostic risk assessment tool was developed to facilitate clinical translation of research findings, providing evidence-based support for precision management and secondary prevention strategies in Chinese patients with ischaemic stroke.

## Methods

This retrospective cohort study was conducted at the First People's Hospital of Xuzhou. From an initial cohort of 743 patients with confirmed acute ischaemic stroke (AIS), 585 were *included* following rigorous application of inclusion and exclusion criteria. Participants were randomly allocated in a 7:3 ratio to training (*n* = 410) and validation (*n* = 175) datasets. Patients were stratified according to modified Rankin Scale (mRS) scores into unfavourable outcome (mRS≥2) and favourable outcome (mRS<2) groups. The study received ethical approval from the Ethics Committee of the First People's Hospital of Xuzhou [2026-快审-5/2026.01.19].

**Inclusion criteria** comprised: (1) age ≥60 years; (2) complete medical records; (3) diagnosis conforming to the 2018 Chinese Guidelines for Diagnosis and Treatment of Acute Ischaemic Stroke (10). The rationale for restricting the cohort to patients aged ≥60 years was to minimise aetiological and pathophysiological heterogeneity. The aetiology of stroke in younger patients fundamentally differs from the predominant atherosclerotic and cardioembolic mechanisms in older adults. Furthermore, as this study evaluated immune-inflammatory biomarkers (e.g., SII), restricting the age range mitigated the confounding effects of age-dependent baseline immunological variations (i.e., “inflammaging”), ensuring a comparable baseline inflammatory profile within the cohort.

**Exclusion criteria** encompassed: (1) absence of laboratory data within 24 h of admission; (2) unavailable mRS assessment at one-year follow-up; (3) concomitant intracranial malignancy, acute traumatic brain injury, dementia, or other neurological disorders; (4) haematological diseases or severe hepatic or renal dysfunction; (5) severe cardiopulmonary insufficiency; (6) pre-existing severe disability or stroke sequelae affecting National Institutes of Health Stroke Scale (NIHSS) assessment; (7) history of dysphagia or long-term nasogastric tube placement; (8) pre-stroke mRS≥2.

### Statistical analysis

This study was reported in adherence to the Transparent Reporting of a multivariable prediction model for Individual Prognosis or Diagnosis (TRIPOD) guidelines. All analyses were performed using R version 4.5.0. Patients with >5% missing values in candidate variables were excluded during the initial screening. For remaining continuous variables, normal distribution was assessed; normally distributed data were expressed as mean ± standard deviation (x¯ ± s) and compared using independent samples *t*-tests, whilst non-normally distributed data were presented as median [M(Q1, Q3)] and analysed using Mann–Whitney *U* tests. Categorical variables were expressed as percentages and evaluated using *χ*^2^ tests.

### Predictive model development and validation

To strictly prevent data leakage, all data preprocessing, feature selection, and hyperparameter optimisation procedures were conducted exclusively within the training dataset after the initial 7:3 random data partition. Univariate and multivariate logistic regressions were initially performed on the training cohort to screen potential predictors. Subsequently, recursive feature elimination (RFE) was employed to iteratively remove variables with the lowest contribution. Our feature selection intentionally prioritized objective, readily available admission laboratory biomarkers and demographics to ensure the model's immediate clinical applicability at presentation.

Ultimately, ten independent predictors were identified. Ten machine learning algorithms were constructed: logistic regression (LR), support vector machine (SVM), gradient boosting machine (GBM), random forest (RF), extreme gradient boosting (XGBoost), light gradient boosting machine (LightGBM), categorical boosting (CatBoost), adaptive boosting (AdaBoost), neural network (NN), and k-nearest neighbours (KNN). To ensure robust internal validation and mitigate the risk of optimistic performance estimates, hyperparameter optimisation was performed employing a rigorous 10-fold cross-validation repeated 5 times (repeatedcv) exclusively within the training set. Grid search was utilised to define parameter ranges based on established literature and exploratory analyses. The finalized models were subsequently evaluated on the unseen validation dataset.

### Model performance evaluation and selection

Multidimensional performance assessment encompassed: (1) discriminative ability: calculation of area under the receiver operating characteristic curve (AUROC) with 95% confidence intervals, with inter-model comparisons performed using DeLong’s test; (2) calibration: graphical assessment via calibration plots; (3) clinical net benefit: decision curve analysis (DCA) evaluating net benefit across risk thresholds relative to “treat all” and “treat none” strategies.

### Model interpretability analysis

SHapley Additive exPlanations (SHAP) methodology was applied for model interpretation. Grounded in game-theoretic Shapley values, SHAP quantifies the marginal contribution of each feature to model predictions. Global SHAP values identified key risk factors and their relative importance, whilst individual-level SHAP values visualised patient-specific risk composition, providing a theoretical foundation for precision clinical intervention.

### Web-based risk assessment tool development

An interactive risk assessment tool was developed using the Shiny framework, deploying the optimal model as an online risk calculator. The front-end interface comprises ten standardised input fields corresponding to core predictive variables; the back-end integrates the trained, optimised model, enabling real-time data preprocessing, prediction, and results visualisation. The system outputs absolute risk probabilities (0%–100%) for one-year adverse outcomes in AIS patients. Deployed on cloud-based servers, the tool supports barrier-free multi-device access without registration requirements, safeguarding user data privacy.

## Results

The study included 585 patients with AIS, randomly allocated in a 7:3 ratio to training (*n* = 410) and validation (*n* = 175) cohorts. Within the training dataset, patients were stratified according to one-year outcomes into favourable (mRS<2, *n* = 320) and unfavourable (mRS≥2, *n* = 90) prognosis groups. Baseline clinical and laboratory characteristics are presented in Table 1.

**Table 1:** Baseline characteristics comparing favourable and unfavourable outcome groups in the training cohort of patients with acute ischaemic stroke.

Characteristic	mRS<2 (*n* = 320)	mRS≥2 (*n* = 90)	*p*-value
Male [*n* (%)]	193 (60.3)	50 (55.6)	0.49
Smoking [*n* (%)]	118 (36.9)	27 (30.0)	0.28
Hypertension [n (%)]	257 (80.3)	78 (86.7)	0.221
Diabetes mellitus [*n* (%)]	142 (44.4)	49 (54.4)	0.116
Hyperlipidaemia [*n* (%)]	189 (59.1)	48 (53.3)	0.395
Coronary artery disease [*n* (%)]	135 (42.2)	44 (48.9)	0.311
Atrial fibrillation [*n* (%)]	23 (7.2)	11 (12.2)	0.189
Internal carotid artery stenosis [*n* (%)]	106 (33.1)	29 (32.2)	0.973
Age [years, mean (SD)]	72.03 (8.34)	76.81 (9.63)	<0.001
Systolic blood pressure [mmHg, mean (SD)]	145.06 (21.29)	149.11 (22.47)	0.116
Height [m, mean (SD)]	1.66 (0.08)	1.66 (0.07)	0.576
Weight [kg, mean (SD)]	68.62 (10.19)	65.93 (9.01)	0.024
White blood cells [×10^9^/L, mean (SD)]	6.77 (2.12)	7.59 (2.73)	0.002
Haemoglobin [g/L, mean (SD)]	132.52 (18.66)	130.31 (17.66)	0.315
Platelets [×10^9^/L, mean (SD)]	216.08 (58.93)	216.63 (57.32)	0.938
Neutrophils [×10^9^/L, mean (SD)]	4.45 (1.92)	5.42 (2.55)	<0.001
Lymphocytes [×10^9^/L, mean (SD)]	1.77 (1.62)	1.53 (0.72)	0.178
Monocytes [×10^9^/L, mean (SD)]	0.47 (0.46)	0.48 (0.19)	0.953
High-sensitivity C-reactive protein [mg/L, mean (SD)]	5.16 (13.50)	10.21 (18.13)	0.004
Prothrombin time [sec, mean (SD)]	11.69 (1.99)	11.69 (1.09)	0.999
Activated partial thromboplastin time [sec, mean (SD)]	31.26 (3.38)	30.76 (4.04)	0.239
Fibrinogen [g/L, mean (SD)]	3.25 (0.67)	3.62 (1.47)	0.001
D-dimer [ng/mL, mean (SD)]	271.47 (575.49)	459.47 (676.99)	0.009
Potassium [mmol/L, mean (SD)]	3.96 (0.41)	3.93 (0.42)	0.519
Sodium [mmol/L, mean (SD)]	139.76 (3.48)	139.83 (4.41)	0.864
Aspartate aminotransferase [U/L, mean (SD)]	19.25 (12.88)	24.67 (22.68)	0.004
Alanine aminotransferase [U/L, mean (SD)]	19.43 (9.50)	24.36 (17.97)	0.001
Fasting blood glucose [mmol/L, mean (SD)]	7.19 (3.22)	8.07 (3.79)	0.03
Creatinine [μmol/L, mean (SD)]	74.66 (29.43)	77.21 (56.72)	0.566
Uric acid [μmol/L, mean (SD)]	313.24 (87.34)	324.95 (130.81)	0.32
Albumin [g/L, mean (SD)]	42.65 (21.35)	44.31 (39.06)	0.597
Total cholesterol [mmol/L, mean (SD)]	4.38 (1.22)	4.47 (1.85)	0.596
Triglycerides [mmol/L, mean (SD)]	1.50 (0.86)	1.46 (0.79)	0.68
High-density lipoprotein [mmol/L, mean (SD)]	1.10 (0.27)	1.10 (0.29)	0.977
Low-density lipoprotein [mmol/L, mean (SD)]	2.50 (0.91)	2.47 (1.00)	0.782
Glycated haemoglobin [%, mean (SD)]	6.68 (1.40)	6.95 (1.57)	0.123
Prognostic nutritional index [mean (SD)]	51.49 (22.60)	51.96 (38.85)	0.882
Haemoglobin-albumin-lymphocyte-platelet score [mean (SD)]	48.77 (50.48)	40.91 (28.25)	0.158
Systemic immune-inflammation index [mean (SD)]	688.29 (782.40)	953.29 (846.71)	0.006

Data are presented as *n* (%) for categorical variables and mean (SD) for continuous variables. mRS, modified Rankin Scale; SD, standard deviation. Between-group comparisons were performed using *χ*^2^ test for categorical variables and independent samples *t*-test for continuous variables. Statistical significance was set at *p* < 0.05. Significant differences (*p* < 0.05) were observed for age, weight, white blood cells, neutrophils, high-sensitivity C-reactive protein, fibrinogen, D-dimer, aspartate aminotransferase, alanine aminotransferase, fasting blood glucose, and systemic immune-inflammation index.

Regarding demographic distribution, male representation was 60.3% in the favourable outcome group versus 55.6% in the unfavourable outcome group; smoking history was present in 36.9% and 30.0%, respectively. Hypertension prevalence was 80.3% and 86.7% in the favourable and unfavourable groups, respectively, whilst diabetes mellitus affected 44.4% and 54.4%. Patients with unfavourable outcomes were significantly older than those with favourable prognosis (76.81 ± 9.63 years vs. 72.03 ± 8.34 years) and exhibited higher systolic blood pressure (149.11 ± 22.47 mmHg vs. 145.06 ± 21.29 mmHg). Laboratory parameters revealed elevated white blood cell count, neutrophil count, and high-sensitivity C-reactive protein (hsCRP) in the unfavourable outcome group compared with the favourable group (white blood cells: 7.59 ± 2.73 vs. 6.77 ± 2.12 × 10^9^/L; neutrophils: 5.42 ± 2.55 vs. 4.45 ± 1.92 × 10^9^/L; hsCRP: 10.21 ± 18.13 vs. 5.16 ± 13.50 mg/L). Furthermore, the unfavourable outcome group demonstrated higher fibrinogen, D-dimer, aspartate aminotransferase (AST), alanine aminotransferase (ALT), fasting glucose, homocysteine, glycated haemoglobin, and SII levels relative to the favourable outcome group. No significant between-group differences were observed for coronary artery disease, atrial fibrillation, carotid stenosis, platelet count, serum potassium, serum sodium, or lipid profiles. Nutritional indices, including PNI and HALP score, exhibited no notable disparities between groups.

These findings indicate that patients with unfavourable outcomes were characterised by advanced age, higher prevalence of diabetes mellitus and hypertension, and significantly elevated inflammatory and coagulation-related laboratory markers, suggesting these factors may be intimately associated with long-term prognosis in AIS patients.

To enhance predictive performance for unfavourable outcomes within one year of AIS onset, recursive feature elimination (RFE) was employed to screen all candidate variables, ultimately identifying 10 key predictive indicators: age, D-dimer, neutrophil count, white blood cell count, uric acid, SII, fasting blood glucose, high-sensitivity C-reactive protein (hsCRP), fibrinogen, and creatinine. This feature combination demonstrated optimal performance during model training, achieving root mean square error (RMSE) of 0.4392, coefficient of determination (R^2^) of 0.07217, and mean absolute error (MAE) of 0.2809, with low standard deviations across metrics, indicating robust stability and generalisation capacity under different data partitions. These 10 variables were incorporated into the core feature set for subsequent machine learning models, establishing a solid foundation for model construction and performance optimisation.

### Model performance and interpretation

Ten mainstream machine learning models were constructed on the training dataset, and their predictive performance was systematically evaluated. Figure 1A presents ROC curves for all models on the validation set, with the random forest model demonstrating superior performance, achieving an AUC of 0.870 (95% CI: 0.816–0.923), outperforming all other models. Figure 1B shows the calibration curve for the validation set, indicating that the random forest model exhibited good calibration ability. Decision curve analysis (Figures 1C,D) further quantified the clinical net benefit of each model across different risk thresholds. Results demonstrated that within the risk threshold range of 10%–85%, the net benefit curve of the random forest model consistently exceeded those of other models, suggesting greater utility for clinical decision-making in practice.

**Figure 1:**
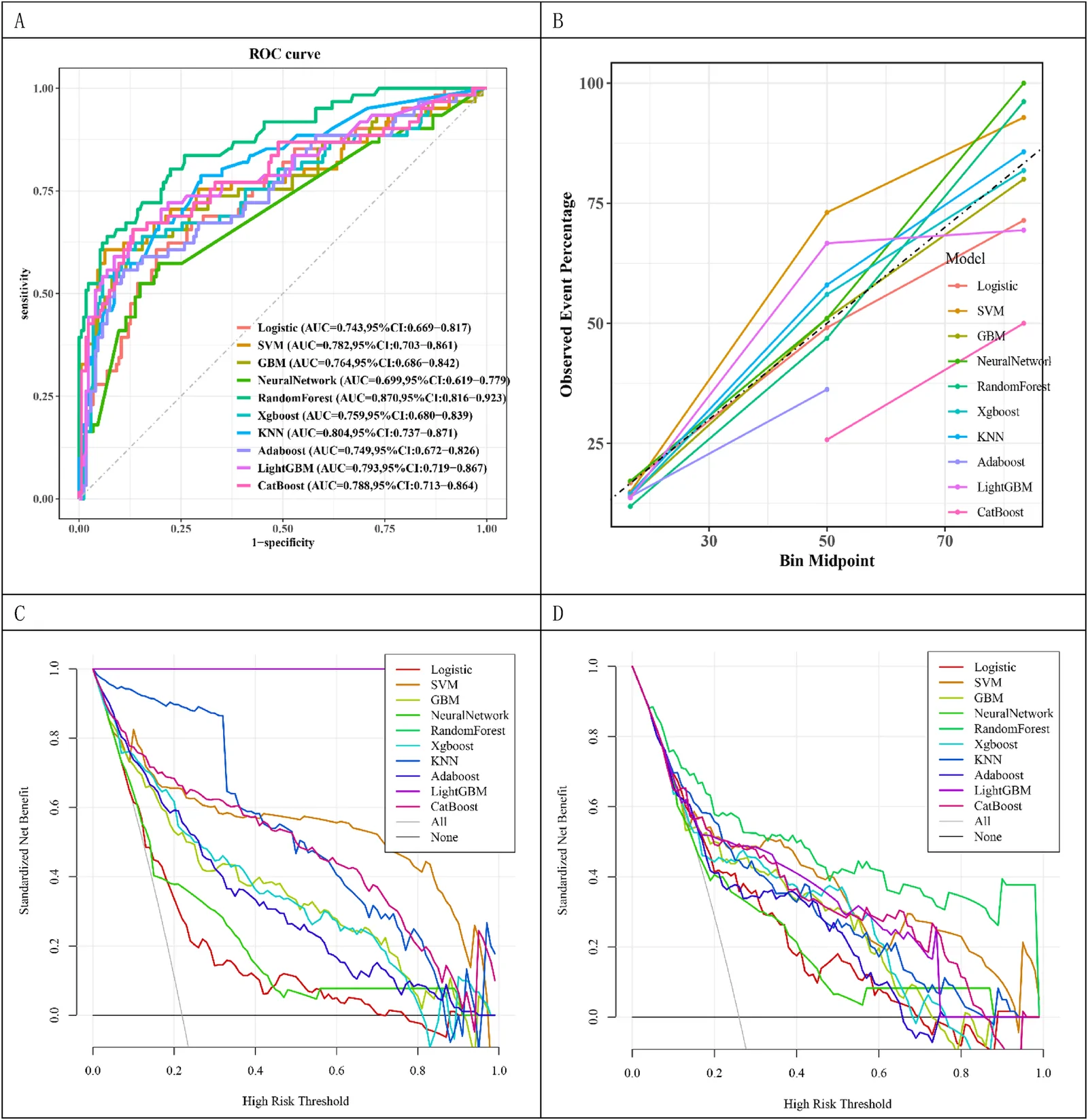
Performance evaluation of the random forest model for predicting one-year unfavourable outcome risk in acute ischaemic stroke.

In further analysis of feature importance within the random forest model, we employed the SHAP (Shapley Additive Explanations) method to visualise the model's decision-making process (see Figure 2). SHAP values quantify both the magnitude and direction of each feature's contribution to model predictions.

**Figure 2:**
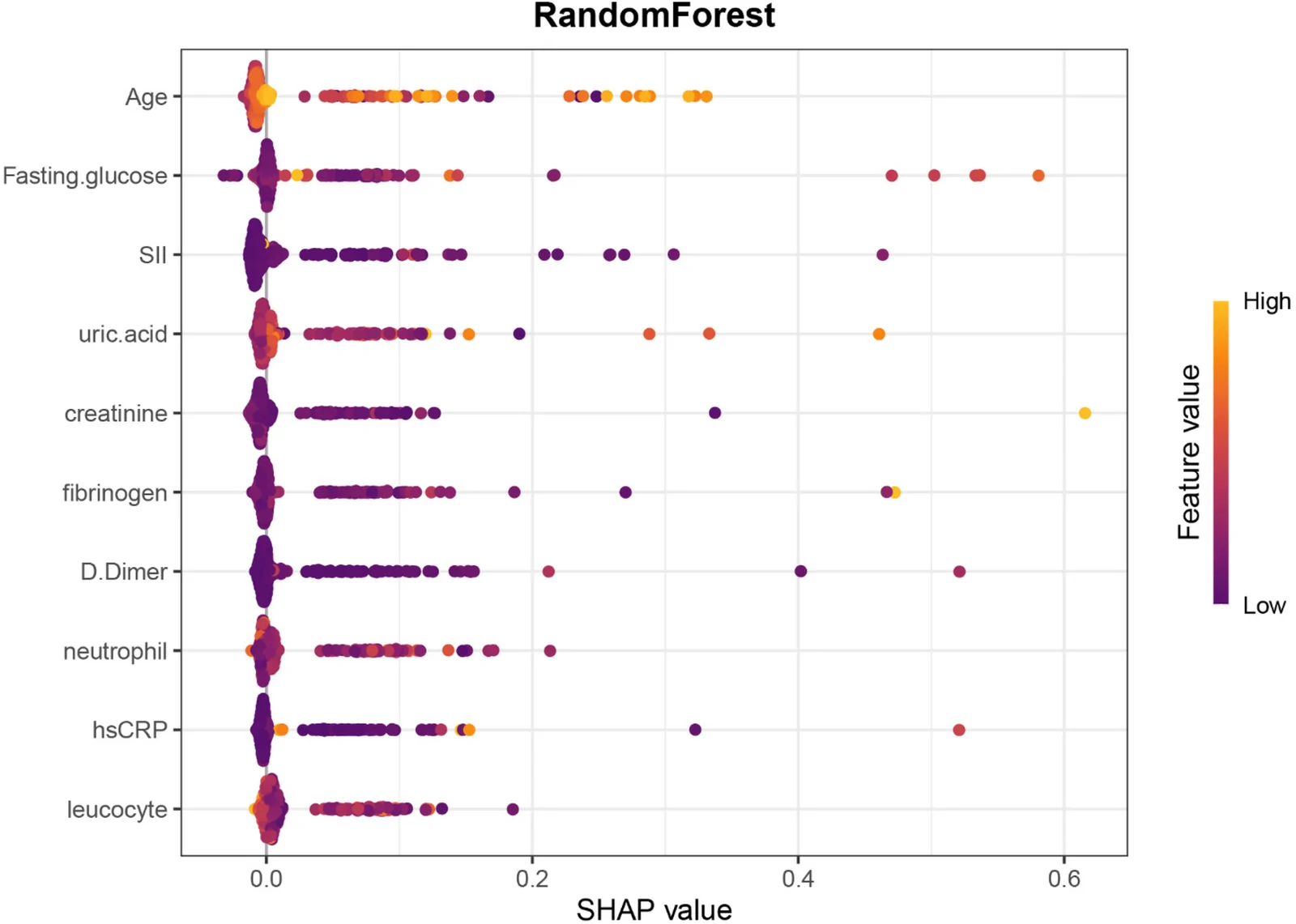
SHAP value analysis of feature contributions in the random forest model for predicting one-year unfavourable outcome risk in acute ischaemic stroke.

The vertical axis displays the principal feature variables incorporated into the model, including age, fasting glucose (Fasting.glucose), SII, uric acid, creatinine, fibrinogen, D-dimer, neutrophil count (neutrophil), high-sensitivity C-reactive protein (hsCRP), and leucocyte count (leucocyte). The horizontal axis represents corresponding SHAP values, whilst the colour bar on the right reflects feature values, with yellow indicating high values and purple indicating low values.

Analysis revealed that high feature values (yellow regions) for age, fasting glucose, fibrinogen, and hsCRP correlated with higher SHAP values, suggesting that elevation of these indicators exerted a significant positive influence on the model's prediction of unfavourable outcomes. Conversely, low values (purple regions) of SII, creatinine, and D-dimer had substantial impact on prediction results, demonstrating their risk-indicating role within the model. Additionally, SHAP value distributions for uric acid, neutrophil count, and leucocyte count were relatively uniform, indicating that both high and low values of these features exerted meaningful influence on prediction results.

In summary, SHAP analysis not only revealed the importance ranking and directional effects of clinical indicators within the random forest model, but also provided data support for understanding model decision mechanisms, optimising risk assessment, and formulating individualised intervention strategies.

### Online risk assessment tool

Based on the validated random forest model, an interactive web-based risk calculator for one-year unfavourable outcome in acute ischaemic stroke was developed and deployed (URL: https://zxy08887.shinyapps.io/1YPP-AIS-Model/). The tool adopts a streamlined two-stage workflow designed to facilitate both clinical risk screening and patient self-assessment.

The interface comprises two functional modules (Figures 3, 4). Figure 3 presents the clinical variable entry interface, structured into three thematic sections: Demographics & Metabolic (age, fasting glucose, uric acid, creatinine), Inflammatory & Immune (leucocyte count, neutrophil count, hsCRP, SII), and Coagulation Profile (D-dimer, fibrinogen). All 10 variables require standardised units as specified in the model training protocol. Upon completion of data entry, users execute risk stratification via a dedicated command button.

**Figure 3:**
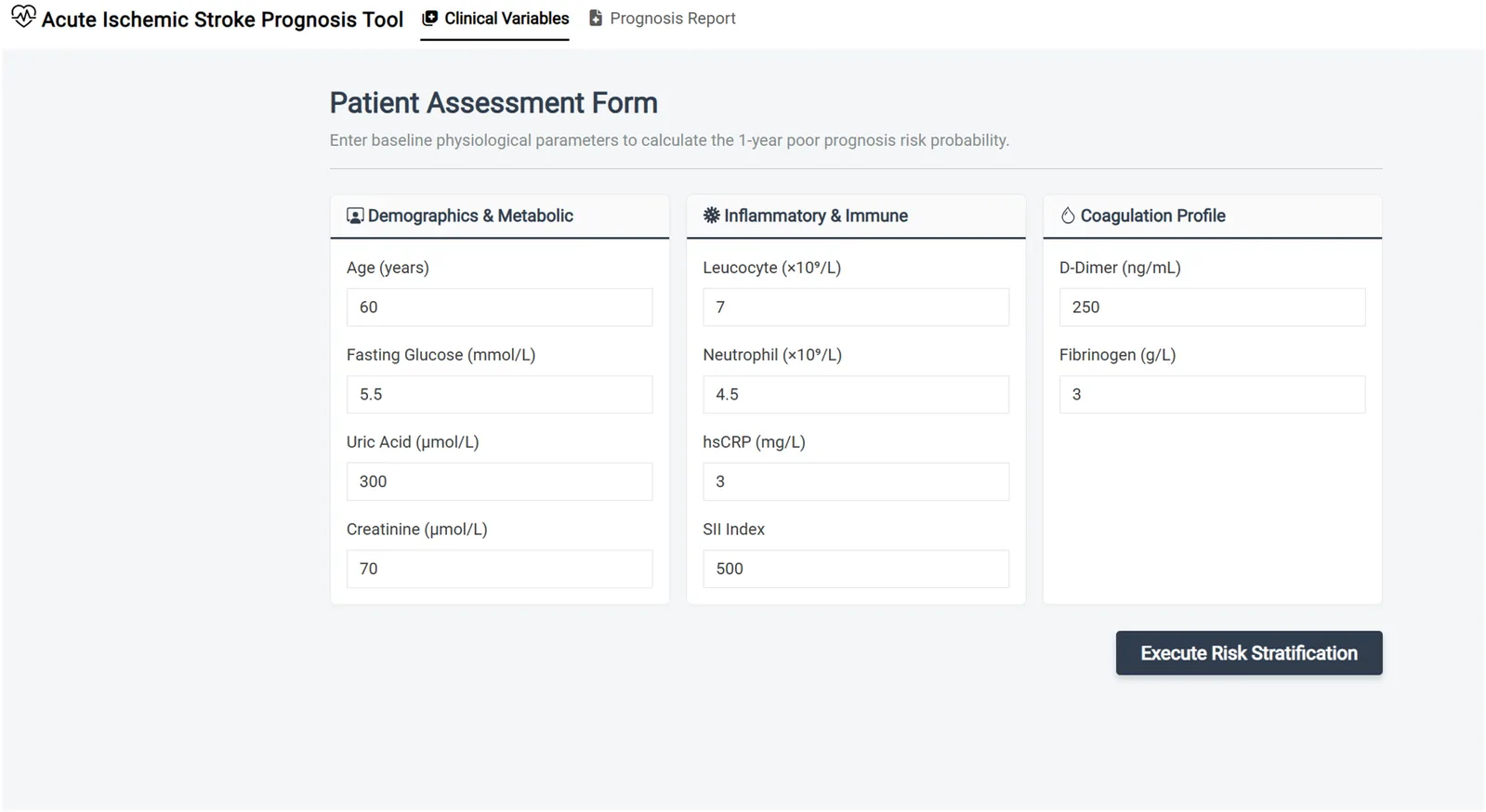
Clinical variable entry interface of the web-based acute ischaemic stroke prognosis tool.

**Figure 4:**
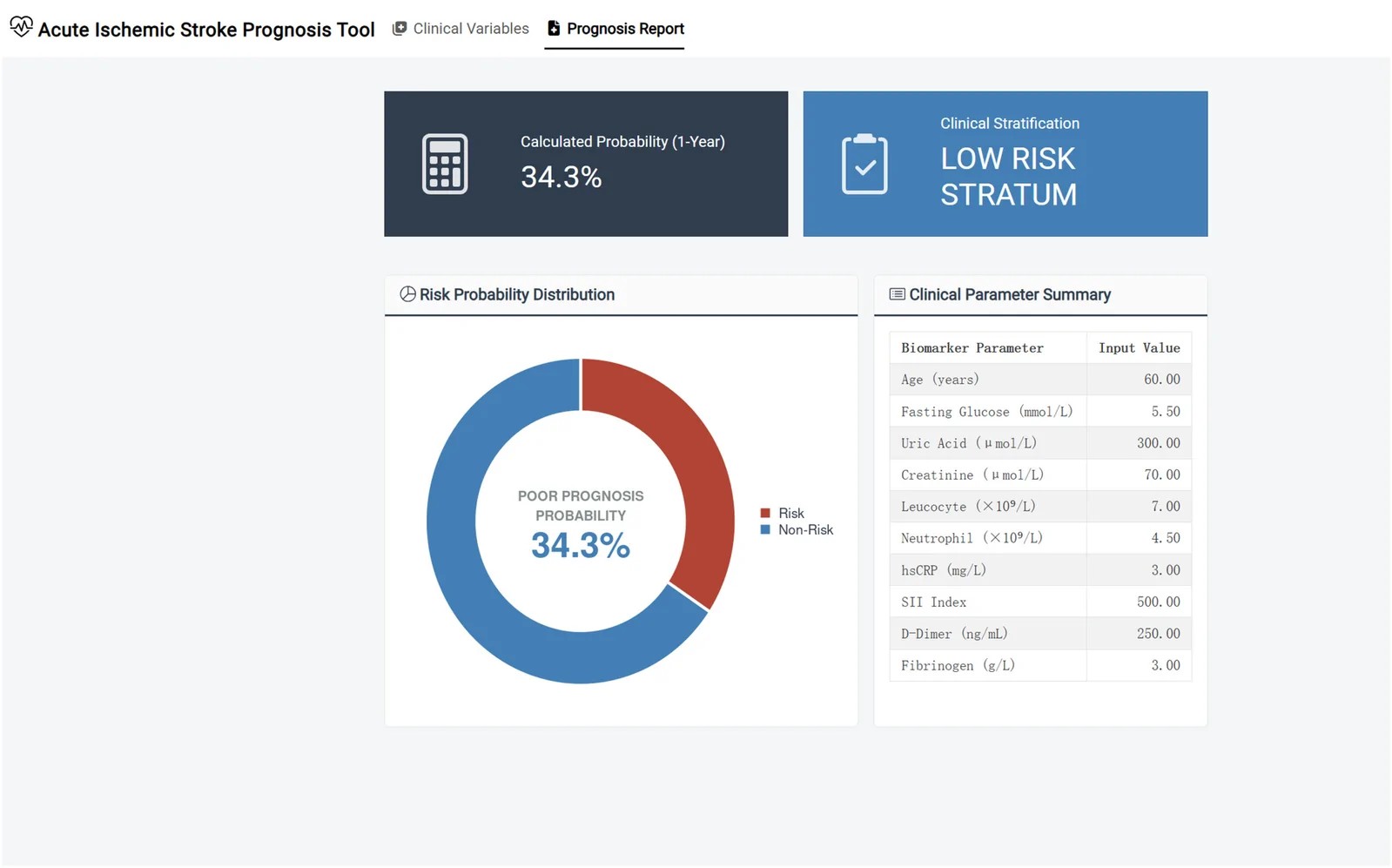
Prognosis report interface displaying calculated risk probability, clinical stratification, and parameter summary.

Figure 4 displays the prognosis report interface, which integrates quantitative and qualitative risk outputs. The calculated one-year poor prognosis probability is presented both numerically and through a proportional ring chart visualising risk versus non-risk distribution. Concurrently, the system assigns clinical stratification (low, intermediate, or high risk stratum) based on pre-defined probability thresholds. A comprehensive parameter summary table is included to enable traceability and verification of input values. The entire computational process operates in real time, providing immediate probabilistic estimates to support evidence-based clinical decision-making and risk communication. Importantly, a clear clinical disclaimer has been embedded within the interface, explicitly stating that the tool currently serves as a research prototype and is not intended to guide direct clinical decision-making prior to rigorous external validation.

## Discussion

Accurately predicting long-term functional outcomes in older adults with acute ischaemic stroke (AIS)—which accounts for the vast majority of all stroke subtypes (11, 12)—remains a formidable clinical challenge due to complex age-dependent baseline comorbidities and distinct inflammatory profiles (13–15). While the widespread adoption of reperfusion therapies has achieved significant progress in promoting functional recovery (16, 17), clinical assessment of post-stroke prognosis is still hampered by the lack of highly accurate and interpretable predictive tools. Developing such tools is crucial for guiding targeted interventions and optimising resource allocation, which ultimately mitigates the societal burden of stroke-related disability (18). To address this clinical gap, this study systematically evaluated and optimised the application of machine learning models to predict the risk of one-year unfavourable outcomes specifically in older AIS patients (aged ≥60 years). Through RFE, we precisely identified key prognostic variables and compared ten mainstream machine learning algorithms, ultimately establishing the random forest algorithm as the optimal model. Our model demonstrated excellent discriminative capacity and calibration in the validation cohort, whilst DCA confirmed substantial net benefit for clinical application. Crucially, the integration of SHAP interpretability analysis effectively elucidated core risk factors and their underlying decision mechanisms, providing a theoretical foundation for precision clinical management and secondary prevention in the older stroke population.

Study findings revealed that age, D-dimer, SII, hsCRP, and fasting glucose exhibited significant predictive value in long-term prognostic assessment of AIS patients. Importantly, the prognostic utility of these acute-phase parameters extends beyond mere statistical association; they fundamentally reflect biological mechanisms that dictate long-term neuro-recovery trajectories. However, it is imperative to note that while SHAP analysis robustly visualizes mathematical feature contributions to the model's predictive output, it does not inherently establish direct causal relationships. Elevated admission D-dimer is not merely an indicator of acute thrombosis but is broadly associated with profound underlying endothelial dysfunction. This persistent microvascular impairment is hypothesized to impede collateral angiogenesis and neurovascular unit remodelling (19). Similarly, acute systemic inflammation, captured by elevated leucocyte counts (within the SII) and hsCRP, correlates with the disruption of the blood-brain barrier and sustained microglial activation. This prolonged neuroinflammation is strongly associated with a neurotoxic microenvironment, which may compromise the secondary synaptic plasticity essential for physical rehabilitation (20). Furthermore, admission hyperglycaemia is known to exacerbate oxidative stress and lactic acidosis within the ischaemic penumbra. When superimposed upon advanced age—which inherently limits neuroplastic reserve—these acute pathophysiological markers synergistically stratify the patient's long-term functional ceiling.

Compared with traditional risk scoring systems and univariate biomarker models, the multivariable machine learning model constructed in this study integrates a broader spectrum of clinical features, comprehensively capturing complex non-linear relationships amongst variables, thereby substantially enhancing predictive accuracy and applicability. Notably, visualisation of model decision-making mechanisms through SHAP methodology further strengthened model transparency and clinical interpretability, facilitating translational application of artificial intelligence technology in stroke management (21). The online risk assessment tool developed based on this model enables individualised, real-time prediction of one-year prognostic risk in AIS patients, providing scientific evidence for clinicians in formulating treatment strategies and follow-up protocols. This facilitates early identification of high-risk patients, optimises resource allocation, and enhances stroke management efficiency.

However, this study possesses certain limitations. Firstly, by restricting the inclusion criteria to patients aged ≥60 years to maintain pathophysiological homogeneity, the generalisability of our machine learning models is inevitably limited to older adult populations. The models’ predictive performance in younger stroke cohorts, who possess distinct aetiologies and baseline inflammatory profiles, remains to be validated. Secondly, as a single-centre retrospective analysis, the model was evaluated using an internal validation cohort randomly split from the same dataset, rendering patient recruitment subject to selection bias. Although we employed rigorous 10-fold repeated cross-validation to mitigate overfitting, this approach may still yield optimistic performance estimates. Rigorous external validation in independent, multicentre cohorts is absolutely essential before this model can be considered for clinical deployment. Thirdly, to ensure the model's utility upon immediate admission, our feature set deliberately relied on demographic characteristics and laboratory biomarkers. Consequently, several critical clinical confounders known to profoundly influence post-stroke trajectories were not captured in our dataset. Notably, established predictors such as initial stroke severity (e.g., baseline NIHSS score)—which establishes the baseline potential for recovery (22)—specific ischaemic stroke subtypes (e.g., TOAST classification), detailed infarct characteristics on neuroimaging, reperfusion therapies (thrombolysis/thrombectomy), and functional status at discharge were omitted. The model also lacks data on severe in-hospital complications, such as hospital-acquired pneumonia, which can critically derail early rehabilitation efforts, as well as specific lifestyle factors, particularly tobacco use—a well-documented driver of poor vascular outcomes (23). The absence of these critical variables raises the necessity for future studies to enhance predictive performance through multimodal data integration, determining whether our biomarker-driven model provides incremental prognostic value over, or should be integrated with, traditional clinical prediction models. Finally, whilst this study employed advanced interpretability methods such as SHAP to enhance algorithmic transparency, practical application still necessitates integration with clinical context and physician expertise to avoid purely mechanistic decision-making.

In conclusion, this study constructed and validated a high-performance, interpretable machine learning model that effectively improved prediction of one-year unfavourable outcomes in acute ischaemic stroke patients, culminating in the development of an online risk assessment tool. These findings provide scientific support for precision risk stratification and individualised intervention in AIS patients, holding substantial significance for advancing intelligent and standardised stroke management. Future efforts should focus on expanding sample size, integrating multicentre and multimodal data, and strengthening clinical implementation of the model to continuously optimise prognostic management of stroke patients.

## Data Availability

The raw data supporting the conclusions of this article will be made available by the authors, without undue reservation.
